# Altered Visual Reliance Induced by Stroboscopic Glasses during Postural Control

**DOI:** 10.3390/ijerph19042076

**Published:** 2022-02-12

**Authors:** Hyunwook Lee, Seunguk Han, Jon Ty Hopkins

**Affiliations:** Department of Exercise Sciences, Brigham Young University, Provo, UT 84604, USA; hyunwook.lee31@gmail.com (H.L.); tyhopkins@byu.edu (J.T.H.)

**Keywords:** sensorimotor system, visual contribution, postural stability

## Abstract

Little is known about how disrupted vision affects visual reliance during postural control. postural control. Twenty-four physically active adults volunteered to participate in the study. Static postural control was quantified with center of pressure measures during a one-legged balance test with four different visual inputs (eyes-open (EO), high frequency of strobe vision (HSV), low frequency of strobe vision (LSV), and eyes-closed (EC)) and on two different surfaces (firm and foam). Dynamic postural control was calculated by the dynamic postural stability index and the Y-Balance test for three different visual inputs (EO, HSV, and LSV) and the two different surfaces. Romberg ratios (HSV/EO, LSV/EO, and EC/EO) were then calculated and used for statistical analysis to assess visual contribution during postural control. In the results, Romberg ratios were higher when people were on the foam surface than the firm surface in center of pressure total path in medial-lateral and anterior-posterior directions (*p* < 0.05, both directions). Similarly, Romberg ratios were higher on the foam surface than the firm surface in dynamic stability index in medial-lateral and anterior-posterior directions (*p* < 0.05, both directions). Stroboscopic glasses could alter visual reliance when the somatosensory system is disturbed by a foam pad during both static and dynamic postural control. Clinicians could use the glasses to manipulate visual reliance during dynamic balance training for patients with musculoskeletal injuries.

## 1. Introduction

Three main sensory systems (somatosensory, visual, and vestibular) contribute to human postural control [1]. In order to maintain balance, complex sensorimotor transformations are needed to integrate several sensory inputs and systemize motor outputs [2]. Additionally, these three sensory systems are able to compensate for each other if one or two of them lose their orientation information [3]. The loss of sensory information occurs during eye closure, or low-light conditions, and perturbed positions, which make people vulnerable to injuries in changing environments [4].

When one or more of these sensory systems is altered, the central nervous system can shift its reliance to more reliable information sources among these sensory systems to control posture. For instance, visual reliance can be increased for people with an impaired somatosensory system due to injuries. Specifically, individuals with chronic ankle instability (CAI) and anterior cruciate ligament (ACL) injuries show higher visual reliance than healthy controls [5,6], which increases the risk of further injuries [7]. Due to the increase in the risk of injuries, previous studies have tried to alter visual reliance for people with musculoskeletal injuries as they applied visual disruption via the eyes-closed condition during balance training. However, one meta-analysis revealed that traditional balance training could not alter visual reliance [8]. In order to alter visual reliance during balance training, based on recommendations from the meta-analysis paper, visual disruption should be applied during more demanding movements, which was limited in traditional balance training because human movement is extremely limited with closed eyes [8]. Therefore, this study used stroboscopic glasses to provide visual disruption during both static and dynamic postural control.

In order to appreciate contributions of sensory information during postural control, both the somatosensory and visual systems should be disrupted [2]. For visual disruption, in this study, stroboscopic glasses were used. Using liquid crystal technology, the lens of the stroboscopic glasses flicker between clear and opaque intermittently, removing visual information [9]. In addition, the glasses may provide effective mechanisms, during physical rehabilitation, to disrupt visual stimuli during dynamic movements. There are several ways to perturb somatosensory input including the use of a foam pad, a roller board, or an unstable surface [7]. In this study, we used a foam pad to disturb the somatosensory system. One study assessed the capability of the stroboscopic glasses to alter visual reliance during single-leg balance [7]. However, the study could not confirm whether visual reliance is altered by stroboscopic glasses since there was no direct comparison between surface conditions. In addition, they only used one level of strobe difficulty during static balance. Therefore, it is imperative to identify whether different levels of strobe difficulty and/or different tasks, such as dynamic stability, affect visual reliance.

Therefore, the purpose of this study was to determine how the stroboscopic glasses and surface condition affect visual reliance in relation to different levels of strobe difficulty during static and dynamic postural control. We hypothesized that visual reliance represented by Romberg ratios can be increased when the somatosensory and/or visual system is disturbed. In addition, the visual reliance would be increased as strobe difficulty increases. If stroboscopic glasses can alter visual reliance when the somatosensory system is disturbed during postural control, we would encourage clinicians to use the glasses for their future rehabilitation programs for patients with musculoskeletal injuries who have higher visual reliance.

## 2. Materials and Methodology

### 2.1. Participants

A total of 24 physically active males and females were recruited from a university population, aged 18–35 years. A feasible sample size of 22 participants was determined by a power analysis, a priori, using previous data with alpha, beta, and Cohen’s d values of 0.05, 0.2, and 0.93, respectively. We limited participant’s age to 35 years to reduce the potential effects of age-related confounding factors that are present in an elderly population, such as muscle weakness, joint pain, structural changes, gait alterations, etc. Participant exclusion criteria included a history of lower limb surgery, fracture, or neurological disorders in their lifetime and any sports-related injuries within the previous 3 months. Subject demographic information is shown in Table 1.

All participants were healthy, as was defined using self-reported disability questionnaires including the Foot and Ankle Ability Measure–Activities of Daily Living and–Sports. Specific participant inclusion criteria for healthy controls included (i) no previous ankle sprain injury, (ii) a score of 100% on the Foot and Ankle Ability Measure–Activities of Daily Living, (iii) a score of 100% on the Foot and Ankle Ability Measure–Sports, and (iv) a history of physical activity at least 3 days/week for a total of 90 min/week in the previous 3 months [10]. All participants provided informed consent prior to their participation, and the study was approved by the Brigham Young University’s institutional review board (F19-107).

### 2.2. Experimental Procedures

Investigators fully reviewed the procedures with each participant, and participants read and signed informed consents prior to data collection. They changed their clothes into spandex shirts, pants, and shoes provided by the investigators. There were three different balance tests including static postural control measured by a one-legged balance test, and dynamic postural control quantified by the dynamic postural stability index (*DPSI*) and the Y-Balance Test (YBT). The static postural control was measured by having subjects stand one-legged on a force plate (AMTI Corp., Watertown, MA) for 5 s with their arms crossed shoulder to shoulder and repeating it three times. They performed the static balance test on the two different surfaces (with and without a foam pad) in four different visual conditions (eyes-open (EO), high frequency of strobe vision (HSV), low frequency of strobe vision (LSV), and eyes-close (EC)). The surface and visual conditions were randomized. Based on the manufacturer’s guideline, HSV was level 2 (4 Hz) and LSV was level 5 (2.25 Hz). To measure *DPSI*, subjects jumped 50% of their maximum jump height 70 cm apart from a force plate where they landed. In order to identify if subjects jumped 50% of their maximum, we set a bar that indicated the 50% of the maximum jump height with a Vertec (Jump USA, Sunnyvale, CA, USA). When subjects touched the bar, we called it a successful jump. After landing, they tried to stabilize as soon as possible. For the YBT, participants performed the test barefoot with the foot positioned and aligned on a slightly elevated block, and then the subjects were instructed to perform the maximal reach distance with the opposite limb by pushing a sliding block using their toes. Each subject performed 4 practice trials in 3 directions (anterior (A), posteromedial (PM), and posterolateral (PL)) on the tested limb. The dynamic postural control, quantified by *DPSI* and YBT were measured on the two different surfaces (with and without a foam pad) in three visual conditions (EO, HSV, and LSV). Participants did not perform the dynamic balance tests under the EC condition due to risk of injuries.

### 2.3. Data Processing

For the static postural control, total path of center of pressure for the medial-lateral (CoP-ML) and anterior-posterior (CoP-AP) directions, and the area of the 95% confidence ellipse of CoP total path were calculated. The *DPSI* was computed using previously described methods [11]. There were three stability indices (SI) based on directions (*MLSI*, *APSI*, and vertical SI (*VSI*)) and *DPSI*. The *MLSI* and *APSI* were defined as the fluctuations from baseline (a zero point) along the frontal and sagittal axes of the force plate, respectively. In other words, the directional indices are mean square deviations assessing fluctuations around a zero point. *VSI* assesses the fluctuation from the subject’s body weight to standardize the vertical ground reaction force along the vertical axis of the force plate. The *DPSI*, as a composite of the other three SIs, is sensitive to changes in all three directions. The following equations were used to calculate the SIs.

$$MLSI=\sqrt{\frac{\sum {\left(0-x\right)}^{2}}{\#\;of\;data\;points}}$$

$$APSI=\sqrt{\frac{\sum {\left(0-y\right)}^{2}}{\#\;of\;data\;points}}$$

$$VSI=\sqrt{\frac{\sum {\left(mass-z\right)}^{2}}{\#\;of\;data\;points}}$$

$$DPSI=\sqrt{\frac{\sum {\left(0-x\right)}^{2}+\sum {\left(0-y\right)}^{2}+\sum {\left(mass-z\right)}^{2}}{\#\;of\;data\;points}}$$

The Y-balance test has been used to measure dynamic postural control in patients with CAI and a previous study found the test could detect deficits related to CAI [12]. A longer reach distance normalized by an individual’s leg length represents better postural control. Reach distances were normalized by leg length (anterior superior iliac spine to the distal end of the medial malleolus). Three trials in each of the 3 directions were used for data analysis.

After all the measures were calculated, we calculated the Romberg ratio in each variable to assess visual reliance during the balance tests [13]. The Romberg ratio has been used to assess visual reliance during postural control [14,15]. The ratio was calculated as HSV/EO, LSV/EO, and EC/EO for the static variables and HSV/EO and LSV/EO for the dynamic variables. A higher ratio represents higher contribution of visual information in the variables. It should be noted that the lower ratio in the YBT means higher visual contribution since a higher score in the YBT represents better balance and postural control.

### 2.4. Statistical Analysis

The independent variables were two surfaces (firm and foam) and three different visual condition ratios (HSV/EO, LSV/EO, and EC/EO) for static postural control and two different visual condition ratios (HSV/EO and LSV/EO) for dynamic postural control. The data from all variables followed normal distribution as evidenced by Shapiro–Wilks tests. Two-way repeated measures analysis of variance was used to assess the difference in Romberg rations between surfaces. A Tukey’s Honestly Significant Difference post-hoc test was performed for pairwise comparisons if they had significant interactions from the repeated measures analysis of variance. The experiment-wise type I error rate for all tests was set at *p* < 0.05. Cohen’s d was calculated to give an impression of the effect size (from 0.21 to 0.5, small; 0.51 to 0.8, moderate; 0.8, large). A statistical software (JMP Pro 14, Cary, NC, USA) was used for all analyses.

## 3. Results

### 3.1. Static Postural Control

**Figure 1:**
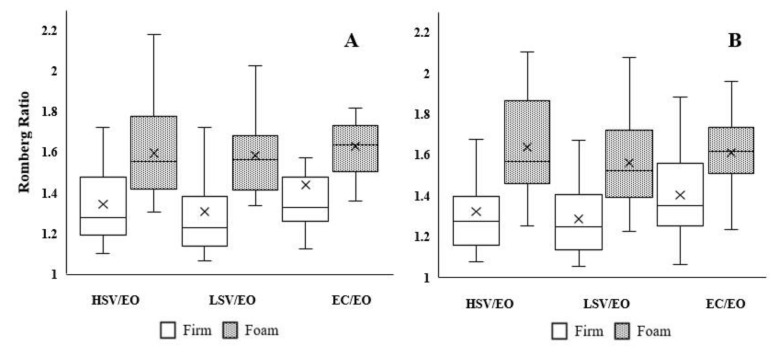
Panel (**A**,**B**) represent CoP-ML and CoP-AP in box plots, respectively. X represents average.

Table 2 represents Romberg ratios with repeated measures analysis of variance and effect sizes during static postural control. We report the values of the static variables in Appendix A. There was no significant interaction in the Romberg ratio for all static variables (CoP-ML: F_5,138_ = 0.57, *p* = 0.56; CoP-AP: F_5,138_ = 0.85, *p* = 0.43; Area of the 95% confidence ellipse of CoP total path: F_5,138_ = 0.43, *p* = 0.65). There were significant main effects of surface condition for CoP-ML (F_5,138_ = 31.79, *p* < 0.0001, d = 0.94) and CoP-AP (F_5,138_ = 58.93, *p* < 0.0001, d = 1.28) (Figure 1). In other words, regardless of the visual condition ratios, the Romberg ratios were higher on the foam surface than the firm surface for CoP-ML (1.60 vs. 1.37) and CoP-AP (1.60 vs. 1.34). Even though large effect sizes were detected between surface conditions in HSV/EO, LSV/EO, and EC/EO for both CoP-ML (0.88, 1.18, and 0.76, respectively) and CoP-AP (1.52, 1.32, and 0.99, respectively), 95% confident intervals crossed 1. There were no significant main effects in surface (F_5,138_ = 0.03, *p* = 0.28, d = 0.32) and vision conditions (F_5,138_ = 1.27, *p* = 0.28) for the area of 95% ellipse of CoP total path (*p* > 0.05).

### 3.2. Dynamic Postural Control

**Figure 2:**
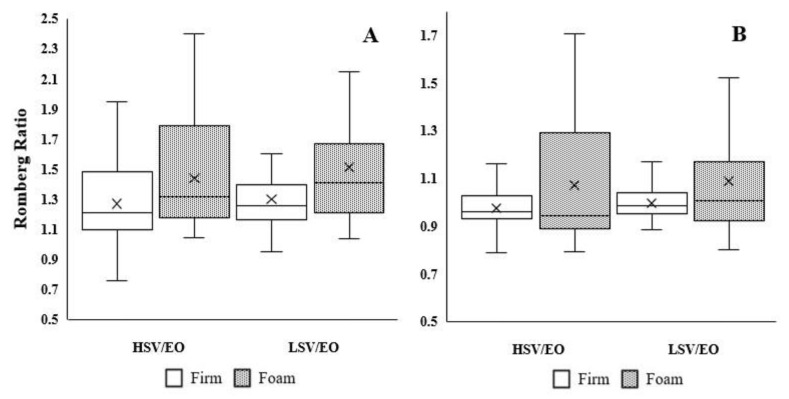
Panel (**A**,**B**) represent medial-lateral and anterior-posterior indices in box plots, respectively. X represents average.

Table 3 represents Romberg ratios with repeated measures analysis of variance and effect sizes during dynamic postural control. We report the values of the dynamic variables in Appendix B. There was no significant interaction in the Romberg ratio for all dynamic variables (*DPSI*: F_3,92_ = 0.12, *p* = 0.71; *MLSI*: F_3,92_ = 0.12, *p* = 0.73; *APSI*: F_3,92_ = 0.01, *p* = 0.94; *VSI*: F_3,92_ = 0.32, *p* = 0.57; YBT-A: F_3,92_ = 0.12, *p* = 0.71; YBT-PL: F_3,92_ = 0.08, *p* = 0.77; YBT-PL: F_3,92_ = 0.27, *p* = 0.60; YBT-PM: F_3,92_ = 0.03, *p* = 0.87). There were significant main effects of surface condition for *MLSI* (F_3,92_ = 8.01, *p* = 0.01, d = 0.58), *APSI* ((F_3,92_ = 6.42, *p* = 0.01, d = 0.51)), and YBT-PM (F_3,92_ = 7.32, *p* = 0.01, d = 0.55) (Figure 2). In other words, regardless of the visual condition, the Romberg ratios were higher on the foam than the firm surface in *MLSI* (1.28 vs. 1.47) and *APSI* (0.99 vs. 1.08). Furthermore, the Romberg ratio was lower on the foam surface than the firm surface in YBT-PM (0.91 vs. 0.96). Even though moderate effect sizes were detected between surface conditions in HSV/EO and LSV/EO for *MLSI* (0.51 and 0.65, respectively), *APSI* (0.53 and 0.50, respectively), and YBT-PM direction (0.59 and 0.52, respectively), 95% confident intervals crossed 1. In addition, there was a significant main effect in visual condition for YBT-PL direction (F_3,92_ = 7.14, *p* = 0.01, d = 0.56). In other words, regardless of surface condition, the Romberg ratio was lower under the LSV condition than the HSV condition in YBT-PL (0.94 vs. 0.99). There were no significant main effects of vision conditions for *DPSI* (F_3,92_ = 0.75, *p* = 0.39, d = 0.17), *MLSI* (F_3,92_ = 0.61, *p* = 0.44, d = 0.16), *APSI* (F_3,92_ = 0.27, *p* = 0.61, d = 0.11), *VSI* (F_3,92_ = 0.53, *p* = 0.47, d = 0.15), and YBT-A (F_3,92_ = 5.13, *p* = 0.03, d = 0.14).

## 4. Discussion

The purpose of this study was to identify the effects of stroboscopic glasses on visual reliance during postural control with different surface conditions and levels of strobe difficulty. The primary finding of this study is that visual contribution was higher on the foam surface than the firm surface during static and dynamic postural control. During the YBT, visual contribution was higher on the foam surface than the firm surface in PM direction. Overall, our results suggest that stroboscopic glasses can alter visual reliance during both static and dynamic postural control.

As far as static postural control, there were significant main effects in the surface condition for CoP-ML and CoP-AP. In other words, the Romberg ratios were higher when participants performed postural control on the foam surface than the firm surface. One study performed similar procedures but did not compare between the surfaces [7]. However, similar to our results, their data indicated that CoP-ML and CoP-AP were faster when their vision was disturbed on the foam surface than the firm surface (EO: 0.79, SV: 1.34, and EC: 1.85 on the firm surface; EO: 1.13, SV: 2.34, and EC: 2.53 on the foam surface). The results might indicate that healthy people rely more on visual information when their somatosensory system and visual information are disturbed by a foam pad and stroboscopic glasses. This assumption can be supported by previous studies that have found increased visual reliance in people with ACL reconstruction and CAI [5,6]. They found out that people with ACL reconstruction and CAI have increased visual information during knee movement [5] and/or postural control [5,6] due to the impaired somatosensory system. They reweight their sensory inputs to the visual system to compensate for loss of and/or decreased sensory information coming from the somatosensory system [5]. Therefore, our results supported that stroboscopic glasses can alter visual reliance when the somatosensory system is disturbed by a foam pad during one-legged postural control.

Similar to the static postural control, the visual contribution was higher in *MLSI* and *APSI* when they were on the foam surface compared to the firm surface. Similarly, visual contribution was higher on the firm surface during the YBT-PM. Since the current study is the first to measure the visual reliance via stroboscopic glasses during dynamic movements, the results could not be compared with previous studies. However, previous studies have consistently reported that visual contribution would be increased when the somatosensory system is disturbed during static postural control [2,7,16,17]. Therefore, this might indicate that stroboscopic glasses could alter visual reliance during dynamic movement as well as during simpler movements, such as one-legged postural control. Since the literature is limited and cannot confirm this assumption, future studies should investigate the effects of stroboscopic glasses on dynamic postural control in patients who have lower extremity pathologies, such as ACL rupture or lateral ankle sprain, to confirm the assumption.

Unlike previous studies measuring only static postural control, the current study examined the effects of stroboscopic glasses on dynamic postural control measured by *DPSI* and YBT. It was limited in explaining visual reliance because of the omission of the EC condition during dynamic postural control due to the risk of injuries. However, previous studies and our results suggested that strobe vision could induce visual disruption as much as the EC condition when the somatosensory system is disrupted [7]. Therefore, we assumed participants might experience a similar level of visual disruption with the EC condition during dynamic postural control with the strobe vision.

As we mentioned in the previous paragraph, our results indicate that visual disruption via stroboscopic glasses could induce visual impairments as much as EC when the somatosensory system is impaired during postural control. This result is aligned with a previous study [7]. It suggests that stroboscopic glasses might be helpful in assessing the extent of reliance on visual information during postural control, as the level of visual disruption can be adjusted until impaired postural control is observed [7]. Furthermore, the ability to modify the level of visual disruption might be able to explain a gap between EO and EC conditions. In the perspective of clinical application, stroboscopic glasses can be widely applied to dynamic balance training, which is a more effective way to improving postural control than static balance training [18].

Unlike previous studies, we added a different frequency of strobe level to examine if the different frequency alters postural control. However, most of the variables showed no statistical difference between the HSV and the LSV conditions during both static and dynamic postural control. As previous studies found, strobe vision and the EC condition could not elicit differences in changing visual reliance during static postural control. Thus, the different strobe levels could not alter the visual contribution during postural control disparately. However, the glasses have eight different levels of visual disruption. We used level 2 (4 Hz of blinking) as a high frequency and level 5 (2.25 Hz of blinking) as a low frequency. Future studies are needed to examine if higher and/or lower frequencies would alter visual contribution during postural control.

It should be noted that even though the results led us to consider visual reliance as one of the superior factors altering balance ability, visual reliance is not the only factor that causes changes in postural control. Based on our results, we could argue that changes in visual reliance occurred when our participants were under various environmental situations induced by stroboscopic glasses and/or uneven surfaces during postural control. It is important to note that the changes could be due to how well the sensory information is integrated at the midbrain or processed at the higher levels and/or simply due to a learning curve on the motor side of the task.

### 4.1. Clinical Implication

One paper, recently published, reported that stroboscopic glasses can induce a degree of postural instability similar to that induced by the sensory organization test (SOT) [16]. The SOT is the gold standard for quantifying sensory dependence by utilizing sway-referenced conditions, but involves expensive equipment [17]. Based on our combined results, we might be able to use stroboscopic glasses, a cost-efficient piece of equipment, rather than the SOT to assess sensory dependence during postural control. Additionally, training with stroboscopic glasses to decrease reliance on visual input for patients with somatosensory deficits could result in improved postural control and general motor function [19]. This disrupted visual feedback could stimulate the neurocognitive demands of activity in a controlled environment. However, clinicians and researchers should be aware that there is a certain discrepancy between stroboscopic glasses-induced and the real world-induced sensory disturbance.

### 4.2. Limitations

The current study has several limitations. First, only a healthy population was recruited for this study. Individuals with lower extremity injuries such as ACL reconstruction and/or ankle sprains might perform differently in terms of visual reliance. Second, the Romberg ratio is just one of the ways to explaining visual contribution during postural control. Future studies are needed to confirm that visual contribution by using imaging tools such as magnetic resonance imaging. Third, current findings can only be generalized to a physically active, college-aged population.

## 5. Conclusions

Physically active and healthy people showed a higher Romberg ratio during static and dynamic postural control when their somatosensory system was disturbed indicating that they might have higher visual reliance. Thus, stroboscopic glasses could alter visual reliance during both static and dynamic postural control. However, the different levels of visual disturbance via the glasses could not elicit differences in visual reliance during the balance tests. We could use stroboscopic glasses not only to identify visual contributions during postural control, but also to improve already-established neuromuscular training methods.

## Figures and Tables

**Table 1:** Participant demographics.

Characteristics	Participants, Mean (SD)
Sex, male/female	12/12
Age, y	21.8 (2.5)
Height, cm	172.0 (7.8)
Mass, kg	73.6 (23.3)
FAAM-ADL, %	0.0 (0.0)
FAAM-Sport, %	0.0 (0.0)

Abbreviations: SD = standard deviation; y = years; cm = centimeter; kg = kilogram; FAAM: foot and ankle ability measure; ADL = activities of daily living.

**Table 2:** Romberg ratios for static postural control with RMANOVA and effect sizes.

	Romberg Ratio, Mean (SD)			RMANOVA			Effect Size, Cohen’s d (95%)
	Visual Condition	Firm Surface	Foam Surface	Surface Main Effect	Visual Condition Main Effect	Interaction	Firm vs. Foam
CoP-ML ^a^	HSV/EO	1.38 (0.31)	1.59 (0.23)	F_5,138_ = 31.79 *p* < 0.0001	F_5,138_ = 1.96 *p* = 0.15	F_5,138_ = 0.57 *p* = 0.56	0.88 (0.30–1.45)
	LSV/EO	1.29 (0.25)	1.59 (0.21)				1.18 (0.59–1.76)
	EC/EO	1.45 (0.26)	1.63 (0.17)				0.76 (0.18–1.33)
CoP-AP ^a^	HSV/EO	1.32 (0.22)	1.64 (0.25)	F_5,138_ = 58.93 *p* < 0.0001	F_5,138_ = 1.99 *p* = 0.14	F_5,138_ = 0.85 *p* = 0.43	1.52 (0.92–2.11)
	LSV/EO	1.29 (0.17)	1.56 (0.21)				1.32 (0.73–1.91)
	EC/EO	1.40 (0.20)	1.61 (0.20)				0.99 (0.42–1.57)
Area of 95% ellipse of CoP total path	HSV/EO	4.59 (2.82)	4.57 (2.38)	F_5,138_ = 0.03 *p* = 0.28	F_5,138_ = 1.27 *p* = 0.28	F_5,138_ = 0.43 *p* = 0.65	0.01 (−5.84–5.86)
	LSV/EO	3.69 (1.99)	4.20 (1.80)				2.42 (−3.45–8.28)
	EC/EO	4.68 (2.09)	4.38 (1.63)				1.45 (−4.41–7.30)

Abbreviation: SD, standard deviation; EO, RMANOVA, repeated measures analysis of variance; eyes open; HSV, high frequency of strobe vision; LSV, low frequency of strobe vision; EC, eyes closed; CoP, center of pressure; ML, medial-lateral; AP, anterior-posterior; ^a^: significant main effect in surface condition (CoP-ML, *p* < 0.0001; CoP-AP, *p* < 0.0001).

**Table 3:** Dynamic postural control: *DPSI* and YBT scores and Romberg ratio.

	Romberg Ratio, Mean (SD)			RMANOVA			Effect Size, Cohen’s d (95%)
	Visual Condition	Firm Surface	Foam Surface	Surface Main Effect	Visual Condition Main Effect	Interaction	Firm vs. Foam
*DPSI*	HSV/EO	1.15 (0.14)	1.19 (0.10)	F_3,92_ = 1.54 *p* = 0.22	F_3,92_ = 0.75 *p* = 0.39	F_3,92_ = 0.12 *p* = 0.71	0.18 (−0.38–0.75)
	LSV/EO	1.16 (0.15)	1.22 (0.08)				0.32 (−0.25–0.89)
*MLSI* ^a^	HSV/EO	1.27 (0.29)	1.44 (0.34)	F_3,92_ = 8.01 *p* = 0.01	F_3,92_ = 0.61 *p* = 0.44	F_3,92_ = 0.12 *p* = 0.73	0.51 (−0.06–1.08)
	LSV/EO	1.30 (0.24)	1.51 (0.42)				0.65 (0.07–1.22)
*APSI* ^a^	HSV/EO	0.98 (0.28)	1.07 (0.25)	F_3,92_ = 6.42 *p* = 0.01	F_3,92_ = 0.27 *p* = 0.61	F_3,92_ = 0.01 *p* = 0.94	0.53 (−0.04–1.10)
	LSV/EO	1.00 (0.27)	1.09 (0.24)				0.50 (−0.07–1.07)
*VSI*	HSV/EO	0.99 (0.10)	0.99 (0.08)	F_3,92_ = 0.28 *p* = 0.59	F_3,92_ = 0.53 *p* = 0.47	F_3,92_ = 0.32 *p* = 0.57	0.01 (−0.55–0.57)
	LSV/EO	0.99 (0.11)	1.02 (0.09)				0.22 (−0.34–0.79)
YBT-A	HSV/EO	0.98 (0.05)	0.98 (0.05)	F_3,92_ = 0.40 *p* = 0.53	F_3,92_ = 5.13 *p* = 0.03	F_3,92_ = 0.08 *p* = 0.77	0.07 (−0.50–0.63)
	LSV/EO	0.96 (0.04)	0.95 (0.05)				0.19 (−0.38–0.75)
YBT-PL ^b^	HSV/EO	1.00 (0.12)	0.98 (0.07)	F_3,92_ = 0.10 *p* = 0.75	F_3,92_ = 7.14 *p* = 0.01	F_3,92_ = 0.27 *p* = 0.60	0.17 (−0.40–0.74)
	LSV/EO	0.94 (0.08)	0.95 (0.07)				0.04 (−0.52–0.61)
YBT-PM ^a^	HSV/EO	0.97 (0.08)	0.92 (0.07)	F_3,92_ = 7.32 *p* = 0.01	F_3,92_ = 1.03 *p* = 0.31	F_3,92_ = 0.03 *p* = 0.87	0.59 (0.01–1.16)
	LSV/EO	0.95 (0.07)	0.91 (0.07)				0.52 (−0.05–1.09)

Abbreviation: SD, standard deviation; RMANOVA, repeated measures analysis of variance; EO, eyes open; HSV, high frequency of strobe vision; LSV, low frequency of strobe vision; *DPSI*, dynamic postural stability index; ML, medial-lateral; AP, anterior-posterior; V, vertical; YBT, Y-Balance test; A, anterior; PM, posteromedial; PL, posterolateral; ^a^: significant main effect in surface condition (*p* = 0.01, all); ^b^: significant main effect in vision condition (*p* = 0.01).

## Data Availability

Not applicable.

## Appendix A

**Table A1:** Average values of static postural control variables.

Variables	Surface	Visual Condition, Mean (SD)			
		EO	HSV	LSV	EC
CoP-ML (cm)	Firm	3.49 (0.57)	4.89 (1.79)	4.60 (1.45)	5.14 (1.61)
	Foam	4.53 (0.92)	7.20 (1.89)	7.16 (1.61)	7.38 (1.45)
CoP-AP (cm)	Firm	3.83 (0.59)	5.07 (1.25)	4.92 (1.00)	5.37 (1.16)
	Foam	5.24 (1.16)	8.57 (1.92)	8.11 (1.45)	8.39 (1.54)
Area of 95% ellipse of CoP path (cm^2^)	Firm	4.56 (2.13)	17.80 (10.04)	14.60 (7.01)	18.74 (7.76)
	Foam	7.51 (3.57)	29.76 (10.92)	27.95 (10.53)	29.17 (8.63)

Abbreviation: SD, standard deviation; EO, eyes open; HSV, high frequency of strobe vision; LSV, low frequency of strobe vision; EC, eyes closed; CoP, center of pressure; ML, medial-lateral; AP, anterior-posterior.

## Appendix B

**Table A2:** Average values of dynamic postural control variables.

Variables	Surface	Visual Condition, Mean (SD)		
		EO	HSV	LSV
*DPSI*	Firm	0.42 (0.04)	0.49 (0.06)	0.51 (0.06)
	Foam	0.50 (0.06)	0.49 (0.07)	0.49 (0.06)
*MLSI*	Firm	0.07 (0.01)	0.11 (0.03)	0.11 (0.03)
	Foam	0.09 (0.02)	0.11 (0.02)	0.12 (0.03)
*APSI*	Firm	0.21 (0.03)	0.24 (0.02)	0.24 (0.03)
	Foam	0.23 (0.03)	0.22 (0.03)	0.23 (0.02)
*VSI*	Firm	0.41 (0.07)	0.42 (0.07)	0.43 (0.07)
	Foam	0.43 (0.08)	0.42 (0.08)	0.42 (0.07)
YBT-A (%/LL)	Firm	0.71 (0.06)	0.69 (0.06)	0.68 (0.06)
	Foam	0.68 (0.08)	0.66 (0.08)	0.64 (0.08)
YBT-PM (%/LL)	Firm	1.13 (0.10)	1.09 (0.12)	1.04 (0.09)
	Foam	1.11 (0.09)	1.02 (0.13)	1.00 (0.08)
YBT-PL (%/LL)	Firm	0.10 (0.05)	0.10 (0.12)	1.07 (0.11)
	Foam	0.99 (0.12)	0.98 (0.14)	0.93 (0.09)

Abbreviation: SD, standard deviation; EO, eyes open; HSV, high frequency of strobe vision; LSV, low frequency of strobe vision; *DPSI*, dynamic postural stability index; ML, medial-lateral; AP, anterior-posterior; V, vertical; YBT, Y-Balance test; LL, leg length; A, anterior; PM, posteromedial; PL, posterolateral.
